# Searching substructures in fragment spaces

**DOI:** 10.1186/1758-2946-3-S1-P11

**Published:** 2011-04-19

**Authors:** H-C Ehrlich, M Rarey

**Affiliations:** 1 Center for Bioinformatics, University of Hamburg, 20146 Hamburg, Germany

## Introduction

Fragment spaces (FSs) are an elegant way to model a large or even infinite number of chemical compounds and their synthetic accessibility. A FS consists of molecular fragments and a set of rules defining how fragments can be combined to products. In virtual screening experiments, FSs might include products with undesired functional groups or inadequate central building blocks. The recognition of such products, especially when they span over multiple fragments, would require their explicit construction from the FS. Due to the generally huge number of possible products in an FS, the complete enumeration is undesired or even impossible. Therefore, algorithms that perform substructure search in FSs must be able to process fragments and joining rules rather than complete molecules. Even though some algorithms that work in FSs exist [1,2], a method that excludes undesired products via substructure definition from a FS is still missing.

## Method

We present and compare two algorithms to modify an FS such that no possible product can include a given functional group or substructure. The methods utilize a search procedure based on the Ullmann [3] respectively the VF2 algorithm [4] for subgraph isomorphism. Thereby, we find substructures that are present inside fragments or would be formed by joining two fragments. After the identification of such fragments, they are either removed from the FS or their joining rules are altered in a way that a formation of the substructure becomes impossible.

## Results

The algorithms are tested on the BRICS fragment space [1]. We exclude substructures described by SMARTS patterns that where collected from literature [5]. The experiments show that the VF2 approach is superior in running time.
